# Immune Evasion of *Mycoplasma gallisepticum*: An Overview

**DOI:** 10.3390/ijms25052824

**Published:** 2024-02-29

**Authors:** Yang Liu, Yongqiang Wang, Shijun J. Zheng

**Affiliations:** 1 National Key Laboratory of Veterinary Public Health Security, Beijing 100193, China; liuyang002383@163.com; 2 Key Laboratory of Animal Epidemiology of the Ministry of Agriculture, Beijing 100193, China; 3 College of Veterinary Medicine, China Agricultural University, Beijing 100193, China

**Keywords:** *Mycoplasma gallisepticum*, inflammation, immune evasion, pathogenesis, immune response

## Abstract

*Mycoplasma gallisepticum* is one of the smallest self-replicating organisms. It causes chronic respiratory disease, leading to significant economic losses in poultry industry. Following *M. gallisepticum* invasion, the pathogen can persist in the host owing to its immune evasion, resulting in long-term chronic infection. The strategies of immune evasion by mycoplasmas are very complex and recent research has unraveled these sophisticated mechanisms. The antigens of *M. gallisepticum* exhibit high-frequency changes in size and expression cycle, allowing them to evade the activation of the host humoral immune response. *M. gallisepticum* can invade non-phagocytic chicken cells and also regulate microRNAs to modulate cell proliferation, inflammation, and apoptosis in tracheal epithelial cells during the disease process. *M. gallisepticum* has been shown to transiently activate the inflammatory response and then inhibit it by suppressing key inflammatory mediators, avoiding being cleared. The regulation and activation of immune cells are important for host response against mycoplasma infection. However, *M. gallisepticum* has been shown to interfere with the functions of macrophages and lymphocytes, compromising their defense capabilities. In addition, the pathogen can cause immunological damage to organs by inducing an inflammatory response, cell apoptosis, and oxidative stress, leading to immunosuppression in the host. This review comprehensively summarizes these evasion tactics employed by *M. gallisepticum*, providing valuable insights into better prevention and control of mycoplasma infection.

## 1. Introduction

*Mycoplasma gallisepticum* is a pathogen that mainly affects chickens and turkeys and causes severe economic losses to the poultry industry across the globe. *M. gallisepticum* infection causes chronic respiratory disease (CRD) and infectious sinusitis in turkeys, affecting feed conversion rate, egg production, hatchability, and mortality in chickens [1,2]. This disease usually develops slowly and leads to chronic infection in poultry. 

*M. gallisepticum* lacks cell walls and is surrounded by a cytoplasmic membrane containing lipoproteins. It does not contain toxins and its lipoproteins play an important role in pathogen invasion, adsorption, and the induction of inflammatory responses and immune escape. In the one to two weeks after *M. gallisepticum* infection, there is an acute phase characterized as immune dysregulation. During this period, mycoplasma lipoproteins activate pattern recognition molecules (PRMs), such as TLR2/4/7/15, inducing the expression of cytokines and chemokines, including C-X-C motif chemokine ligand (CXCL)13, CXCL14, C-C motif chemokine ligand 5 (CCL5/RANTES), and interleukin 1β (IL-1β) [3,4]. Large numbers of lymphocytes infiltrate the tracheal tissues, causing local inflammation, but phagocytosis is not fully activated, especially in the absence of specific antibodies [5,6,7]. Mycoplasma may suppress the immune response to some extent, thus allowing the persistence of the infection. It was found that CD8^+^ T cells were absent in the acute phase [3,8] and that the predominant type of antibody secreted by B cells as well as the amount and pattern of B cell infiltration differed between unvaccinated and vaccinated chickens [3,9,10]. The suppression of the adaptive immune response by *M. gallisepticum* may be crucial to the progression of the disease as local antibodies and cell-mediated immunity are effective in clearing and preventing *M. gallisepticum* infection [11]. Immune evasion, therefore, presents significant challenges to the control and clearance of the pathogen. 

In most cases, immunization of flocks with vaccines is an essential means of the prevention and control of infectious diseases. However, the current vaccines against *M. gallisepticum* provide only limited protection and partial or temporary immunity against *M. gallisepticum* infection. More effective vaccines against *M. gallisepticum* infection are in urgent demand. However, the development of novel vaccines requires guidance from a deep understanding of the strategies employed by *M. gallisepticum* for its survival and immune evasion in the host. In recent years, considerable progress has been made in the understanding of the pathogenesis of *M. gallisepticum* infection. Therefore, this review focuses on our current understanding of the interaction of *M. gallisepticum* with the host, in particular, the immune evasion of *M. gallisepticum* from the host immune response. This information may contribute to the development of potential therapeutic approaches for the control of *M. gallisepticum* infection.

## 2. Invasion and Transmission

Although *M. gallisepticum* is not highly transmissible, it can spread vertically and horizontally and its infection of flocks on farms is difficult to completely eradicate, often persisting for a long time [12]. The mechanism of mycoplasma cell infection can be summarized as gliding, adhesion, and invasion. *M. gallisepticum* lacks flagella or pili but could form a membrane protrusion composed of bleb and infrableb, allowing it to glide [13] and work cooperatively with adhesin complexes, namely CrmA and GapA [14,15]. This gliding ability enables them to reach epithelial surfaces and to breach certain physical defenses of the host such as ciliary activity and the mucin layer in the respiratory tract [16]. 

*M. gallisepticum* has a high affinity towards chicken respiratory epithelial cells by binding to host cells through adhesins, inducing the expressions of a series of pro-inflammatory cytokines and causing apoptosis or necroptosis in cells [7,17,18]. *M. gallisepticum* hemagglutinin protein pMGA1.2 interacts with cellular protein ApoA-I to establish infection [19]. Other surface proteins, such as hemadsorption-mediating proteins GapA and CrmA or variable surface membrane proteins (p30, p48, p50, and p80), are able to adhere to cells and serve as prerequisite adhesion components for cell invasion [20]. GapA, CrmA, and Mgc2 have been shown to be involved in the gliding ability of *M. gallisepticum*, necessary for effective adhesion and movement of the microbe from the cilia tips to the apical cell membrane, leading to cell invasion [21].

*M. gallisepticum* also utilizes extracellular matrix (ECM) proteins as a secondary anchoring system [22] as well as its surface protein PlpA; Hlp3 has been shown to bind to the gelatin/heparin-binding structural domain of fibronectin [23]. It was suggested that the *M. gallisepticum* strain R_high_ [24,25] has reduced cytadherence capacities [20] due to the lack of major cytadherence proteins GapA and CrmA [21], which improves cell adhesion rates in Hela cells when preincubated with plasminogen, fibronectin, and plasmin. It seems that the invading process of mycoplasma is much more complicated than predicted.

Cytadherence is a prerequisite for mycoplasma cell invasion and some studies suggest that mycoplasma entry into cells may be via lipid rafts [22]. However, the mechanism of *M. gallisepticum* is less certain. It was reported that *M. gallisepticum* synthesizes hydrogen peroxide to initiate lipid peroxidation of host cell membrane, thereby compromising the integrity and permeability of the eukaryotic cell membrane and facilitating bacterial entry [26]. Other mycoplasmas are diverse and may involve cellular pathways or endocytic mechanisms [27,28].

*M. gallisepticum* adheres to the tracheal mucosa, causing damage to ciliated cells. This impairs the cell’s ability to expel foreign materials and sticky secretions from the trachea, resulting in lung lesions such as tracheitis and airsacculitis that affect normal breathing. Once the lungs are colonized with *M. gallisepticum*, the invader spreads to all organs of the body and its presence can be detected in the heart, brain, liver, spleen, and kidneys [29]. This may be related to the fact that *M. gallisepticum* invades non-phagocytic chicken cells, such as red blood cells, tracheal epithelium, and embryonic fibroblasts, and reaches the distal limbs through the blood [30,31]. In some cases, *M. gallisepticum* infection is also associated with arthritis [32], salpingitis [33], conjunctivitis [34], and fatal encephalopathy [35]. Thus, *M. gallisepticum* infection could impact multiple organs or tissues, causing considerable economic losses to the stakeholders.

*M. gallisepticum* has acquired a perfect transport system, which benefits from the ability to invade the host’s erythrocytes during infection and cross the mucosal barrier to spread systemically in vivo [31]. This allows *M. gallisepticum* to settle in tissues at a distance while being protected by the host immune system and then possibly escape by lysing the erythrocytes with the help of membrane-bound hemolysin activity [36]. Thus, mycoplasma takes full advantage of host erythrocytes to benefit its survival and spread.

## 3. Genetic and Protein Characteristics of *M. gallisepticum*

Following mycoplasma invasion, the host initiates an immune response that suppresses *M. gallisepticum* infection. However, many mycoplasmas, including *M. gallisepticum*, are able to establish successful infection in a wide range of hosts and often cause chronic disease, even in the presence of a specific immune response. Their successful colonization and survival in hosts may contribute to the ability of pathogens to evade host immunity by rapidly changing the phase and size of antigens on their surfaces. For example, the pMGA gene family of *M. gallisepticum* adhesion proteins produces antigenic variants by switching the expression of different pMGA genes [37,38]. 

Mycoplasmas possess many complex systems that control the mechanisms directed toward the phase and size variation of surface lipoproteins with a high-frequency variation [39,40]. This allows one variant to prevail when faced with unpredictable challenges. So far, two possible mechanisms for the induction of variants have been described: one is based on spontaneous mutations in regions prone to DNA slippage due to nucleotide insertions/deletions in homologous or heterologous polynucleotide tracts or short tandem repeats [41,42], occuring mainly in *M. hyorhinis* and *M. gallisepticum*, and the other involves chromosomal rearrangements, which are utilized, for example, by *M. bovis* and *M. synoviae* [43,44]. It seems that the mechanism for induction of variants of mycoplasma is related to the host to some extent.

**Figure 1:**
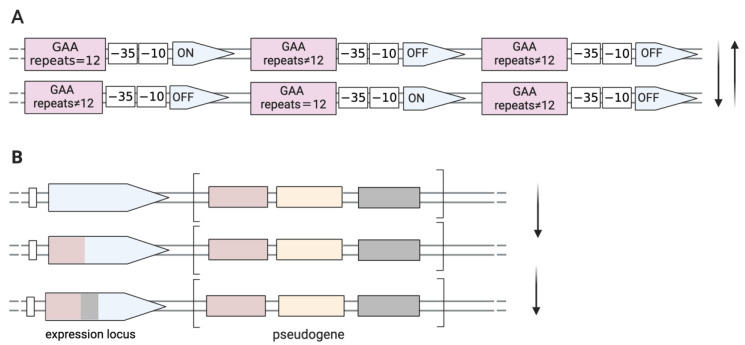
Genetic mechanisms directed towards phase and size variation of surface proteins with a high frequency. (**A**) ON/OFF switching by DNA slippage in *M. gallisepticum*. Spontaneously high-frequency mutations occur in the GAA trinucleotide motifs located upstream of the −35 boxes of the pMGA gene. Transcription of any pMGA gene occurs only when there are twelve GAA trinucleotide repeats. (**B**) Antigenic variations in *M. synoviae* occur through unidirectional recombination (gene conversion) that generates a chimeric-expressed gene, occurring between an expressed locus and pseudogenes, which are the reservoir sequence pools located in relatively closed clusters.

*M. gallisepticum* contains up to 50 members related to genes for putative adhesion molecules (pMGA) [45]. The expression of the pMGA genes of *M. gallisepticum* is controlled by variation in the GAA trinucleotide repeat lengths within the 5′ non-coding regions [42]. As shown in Figure 1, there are 12 GAA trinucleotide motifs upstream of the −35 boxes of the pMGA1.1 gene. The downstream pMGA gene is only expressed when the number of repeats of upstream GAA trinucleotide is 12. Interestingly, the expression of one member of the pMGA gene family is accompanied by the cessation of other pMGA transcription and usually only one pMGA gene is predominantly expressed in any given strain [46]. High-frequency alterations in 5′ trinucleotide repeat numbers are the predominant reason for the switch from producing pMGA1.1 to producing alternative pMGA1.9 on the surface of *M. gallisepticum* strain S6 in vitro culture in the presence of specific antibodies against pMGA1.1 in the growth medium [47]. It was reported that the pMGA phenotype of *M. gallisepticum* could reversibly switch from the expression of pMGA1.1 to pMGA1.2 in vivo infection and this rapid and reversible switch was also detected in vitro when co-cultured with pMGA1.1 antibody. But in vivo, the switching of pMGA protein occurs before host pMGA antibody production, suggesting that factors other than antibodies could influence *M. gallisepticum* population conversion and that changes in the pMGA gene may be necessary for *M. gallisepticum* colonization, infection, or escape from host immunity [38].

## 4. The Innate Immune Response to *M. gallisepticum*

Innate immunity is the first line of host defense against pathogenic infection and a strong inflammatory response is closely associated with *M. gallisepticum*-caused CRD [48]. *M. gallisepticum* attaches and colonizes at the respiratory epithelium and these events occur nearly without classical invasion of tracheal epithelial cells or breach of the mucosal barrier. However, it can mediate severe inflammation and a marked subepithelial infiltration initially by heterophils and macrophages, followed by infiltration of lymphocytes, including B and T cells, into the mucosa [49]. Macrophages and other inflammatory cells are recruited into tracheal mucosa along with the upregulation of chemokines, inflammatory factors, and Toll-like receptor (TLR)-mediated signal activation, including IL-1, IL-8, CXCL13, RANTES, and macrophage inflammatory protein-1β (MIP-1β/CCL4) [4], subsequently recruiting and activating naive B and T lymphocytes into tissues, resulting in more profound dysregulation of the subsequent adaptive immune response. 

After *M. gallisepticum* infection, it was found that the expression of cytokines was upregulated in samples, irrespective of whether the specimens were taken from whole tracheal structures [4] or lavage fluid [6]. A rapid host response was observed 24 h after infection with *M. gallisepticum*, with more than 2500 differentially expressed genes on the peak day of infection, followed by a gradual decrease in differences [50]. Many of these genes are involved in immune-related signaling pathways, including TLR, the mitogen-activated protein kinase (MAPK) pathway, Janus kinase/signal transducer and activator of transcription (Jak-STAT), the NOD-like receptor signaling pathway [50], injury response, metabolism, cell adhesion and remodeling, extracellular matrix (ECM) degradation, and membrane transport [6]; the gene downregulation is associated with impaired ciliary body movement and intercellular connections in infected *M. gallisepticum* strain Ap3AS [5], which ultimately contributes to the pathological damages of the epithelial barrier.

TLRs play important roles in inflammation and innate immune response [51], the expression of many cell surface receptors increased post mycoplasma infection, such as TLR2, TLR4, and TLR15 [50] as well as TLR7 [52]. Their expressions may involve severe immune dysregulation and the development of diseases. TLRs could recognize multiple components of pathogens, including bacteria, viruses, fungi, and protozoa [53]. Bacterial lipoproteins serve as pathogen-associated molecular patterns (PAMPs) that are recognized by the heterodimerization of TLR1/2 or TLR2/6 [54,55]. Most mycoplasma lipopeptides are diacylated and recognized by TLR2/6 and triacylated lipopeptides of mycoplasma are recognized by TLR1/2 [56]. Additionally, TLR15 also recognizes *M. synoviae* diacylated lipopeptide based on the N-terminal sequence of MSPB [57]. Recognition of PAMPs by TLRs activates the NF-κB-mediated cascade inflammatory responses, leading to the expression of proinflammatory cytokines, such as IL-1β, IL-6, IL-8, and tumor necrosis factor alpha (TNF-α) [58], which are associated with inflammatory lung injury [59].

*M. gallisepticum* infection triggers the nuclear factor kappa B (NF-κB) signaling pathway through TLRs including TLR-2/TLR-4/TLR-6 and nod-like receptors (NLRs) [60,61,62]. It was reported that *M. gallisepticum* -HS infection upregulates TLR6 and stimulates IL-2/IL-6-mediated inflammatory responses in DF-1 cells via the TLR6-MyD88-NF-κB signaling pathway [60]. *M. gallisepticum* lipid-associated membrane proteins (LAMPs) induce the expressions of IL-1β, IL-6, IL-8, IL-12p40, CCL-20, and nitric oxide synthase-2 (NOS-2) in epithelial cells when treated both in vitro and ex vivo [63]. It was found that *M. gallisepticum* LAMPs-induced IL-1β expression involved the NF-κB signaling pathway via TLR2 in DF-1 cells and chicken tracheal epithelial cells [64]. IL-12p40 mRNA is usually up-regulated but declined rapidly thereafter [63]. The inflammatory response is triggered by *M. gallisepticum* infection in macrophages via the TLR-2-NF-κB-mediated NLRP3-inflammasome pathway [65]. TLR2 can also activate autophagy through the extracellular signal-related kinase (ERK1/2) signaling pathway, which helps resist *M. gallisepticum* invasion. Although *M. gallisepticum* does not possess lipopolysaccharide (LPS), it can increase the expression of TLR4, which is possibly due to increased numbers of immune cells [66,67]. Recently, it has been found that damage-associated molecular pattern (DAMP)-family molecules, such as high-mobility group box 1 (HMGB1), act as inflammatory mediators secreted by infected macrophages, which could stimulate nearby immune cells such as monocytes, dendritic cells, and monophagocytes [68]. Extracellular HMGB1 upregulates the expression of pro-inflammatory factors including IL-6, IL-1β, IL-12, and TNF-α through the activation of TLR2/4, causing cytokine storms, immune disorders, and severe apoptosis in the spleen, thymus, and bursa of Fabricius [69].

In general, the infection of chickens with *M. gallisepticum* sensed by TLRs triggers the NF-κB signaling pathway, causing inflammatory responses. If the inflammatory response is uncontrollable, excessive proinflammatory cytokines, such as IL-6, IL-1β, IL-12, and TNF-α would be produced, resulting in cytokine storms associated with immune damage or even sepsis. Although inflammation is necessary to combat the spread of microbes, excessive inflammation can still cause tissue damage and is one of the key features of *M. gallisepticum*-induced CRD [70]. However, suppression of the innate immune response may have exacerbated *M. gallisepticum* infection [71]. This will be discussed in detail in the following section (Section 5).

## 5. Escape from Innate Immune Response 

### 5.1. Regulation of the Inflammatory and Chemokine Cytokines

The inflammatory response is modulated by a number of transcription factors and cellular pathways [72], which promote the production of ROS and pro-inflammatory mediators [73]. Some key inflammatory cytokines, such as TNF-α, IL-1 and IL-6, are capable of initiating cell recruitment to facilitate the clearance of pathogens [74]. It was demonstrated using in vitro assays that *M. gallisepticum* is able to interfere with the inflammatory response. IL-6 and TNF-α did not change significantly up to 8 days after *M. gallisepticum* strain R_low_ infection, except that IL-1 was significantly up-regulated at 3 days post-infection [4]. Similarly, the expression of the tumor necrosis factor (TNF) gene and the CCL20 gene was downregulated two weeks after infection with *M. gallisepticum* strain Ap3AS [5], which may be attributed to the immunomodulatory effects of *M. gallisepticum* [4]. The mRNA levels of IL-1β, IL-6, and CCL20, which are known to trigger an inflammatory response, peaked at 6 h following infection with *M. gallisepticum* in HD-11 cells and tracheal epithelial cells, then gradually decreased and reached the baseline 24 h post-infection [7]. Eight hours after *M. gallisepticum* infection in chicken primary alveolar type II epithelial cells, large amounts of TNF-α and IL-6 were produced and inflammation was suppressed 24 h after infection [71]. *M. gallisepticum* causes a strong inflammatory response but does not stimulate certain classical cytokines and it has been shown to transiently activate and then inhibit the inflammatory response [75], which may be one of the reasons for immune dysregulation and evasion of premature clearance by the organism. In addition, several studies suggest that the functions performed by macrophages are not fully activated because TNF-α and IL-12 tended to be downregulated [4,5,6]. Phagocytosis by the macrophage could be observed 2 weeks post-infection, after the phase of immune dysregulation [5]. The interaction of *M. gallisepticum* with host respiratory epithelial cells prompts macrophage chemotaxis and activation, which leads to the expression of a unique set of inflammatory and chemokine genes. However, key cytokines such as IL-12p40 failed to be up-regulated, which may lead to incomplete macrophage activation and thus reduced mycoplasma clearance efficiency [7]. Recent studies have shown that extracellular vesicles (EVs) can influence the downregulation of inflammatory factors and the activation of macrophages in vitro. It has been observed that while *M. gallisepticum* infection alone upregulates pro-inflammatory factors such as TNF-α, IL-1β and IL-6, the presence of *M. gallisepticum*-derived EVs results in a dose-dependent suppression of these factors in HD11 cells at 24 h post-infection [76].

Mycoplasmas can regulate the inflammatory response by activating the nuclear factor erythroid 2-related factor-2 (Nrf2) [77,78]. The lipopeptide MALP-2 (macrophage-activating lipopeptide) from *M. fermentans* is the first lipopeptide known to bind Toll-like receptors (TLR2/6) [79,80]. Its activation leads to an increase in the expression of heme oxygenase-1 (HO-1) [81], an enzyme involved in cellular defense against oxidative stress [82]. By upregulating HO-1, the release of pro-inflammatory molecules such as IL-6, IL-8, ROS, NO, and PGE2 was negatively regulated in the *M. pneumoniae* LAMPs-stimulated cells, thereby helping Mycoplasma evade the immune system and suppress the overall inflammatory response [77,83]. In vitro experiments reveal that the membrane lipoproteins (LAMPs), along with lipoprotein derivatives (lipopeptide MALP-2) in mycoplasmas, induce a “cross-talk” between the pro-inflammatory and anti-inflammatory signaling pathways, including NF-κB and Nrf2/ARE [83]. A homolog of macrophage-activating lipoprotein (MALP-2) in *M. gallisepticum* has been characterized and was found to have no effect on attachment, growth, or pathogenicity in tracheal organ cultures [84] and the exact role of P47 in regulating inflammation is still unclear. Moreover, Nrf2 and HO-1mRNA and protein expression levels did not significantly change in the *M. gallisepticum* infected group compared to the control group [85]. Overall, mycoplasmas may interfere with the inflammatory response to evade the immune system but the exact mechanism requires further study.

### 5.2. Effects of M. gallisepticum at an RNA Level

**Figure 2:**
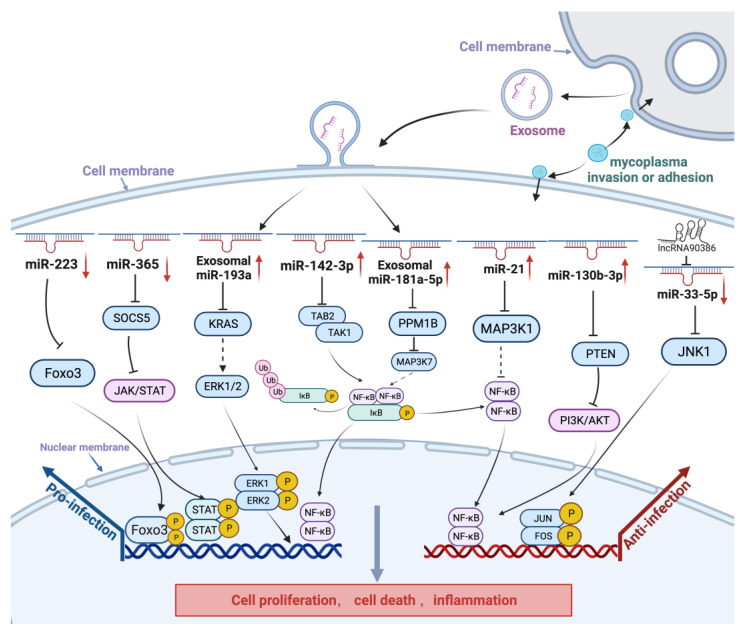
Schematic diagram of non-coding RNAs in the host response to *M. gallisepticum* infection. Upward arrows indicate an increase in the expression of miRNAs, while downward arrows denote a decrease in their expression. After *M. gallisepticum* infection, host cells inhibit *M. gallisepticum* replication, for example, by regulating the expression of miRNAs (e.g., miR-181a-5p, miR-21, and miR-130b-3p) to promote cell proliferation, inhibit cell apoptosis, and enhance the expression of inflammatory cytokines. In addition, *M. gallisepticum* can regulate miRNAs (e.g., miR-223, miR-365-3p, miR-193a, and miR-142-3p) to promote self-replication. In the early stage of *M. gallisepticum* infection in cells, gga-miR-365-3p was upregulated and activated the JAK/STAT signaling pathway to suppress the expression of pMGA1.2. At the later stage of infection, gga-miR-365-3p was downregulated through a negative feedback mechanism, thereby promoting mycoplasma adhesion or invasion.

MicroRNAs (miRNAs) are small noncoding RNAs of 20 to 24 nucleotides that regulate eukaryotic gene expression post transcriptionally by affecting the degradation and translation of target mRNAs [86]. They can bind to the mRNA 3′UTR to promote mRNA degradation or inhibit translation [87]. It was reported that respiratory infectious diseases are closely related to miRNAs [88,89]. After *M. gallisepticum* infection, miRNAs were differentially expressed in the lungs of chicken embryos, with 36 down-regulated and 9 up-regulated miRNAs belonging to a family of 31 miRNAs detected at 3 days post-infection, while 50 down-regulated and 18 up-regulated miRNAs were found at 10 days post-infection. These altered miRNAs target genes in diverse pathways, such as the MAPK pathway, focal adhesion, Wnt pathway, endocytosis, Jak/STAT pathway, phosphatidylinositol pathway, and adherens junctions [62]. Additionally, about 30 microRNAs derived from CP-II cell-derived exosomes were significantly differentially expressed post *M. gallisepticum* infection. Those exosome-miRNAs exerted influence on both neighboring and distal cells by regulating cellular pathways, particularly in the cell cycle, Toll-like receptor signaling pathway, and MAPK signaling pathway [90]. Furthermore, miRNAs play a pivotal role in regulating these cellular processes, which in turn impacts the replication of *M. gallisepticum*, as illustrated in Figure 2.

It has been found that *M. gallisepticum* infection inhibits the cell cycle by blocking the transition of DF-1 cells from G1 to S and G2 phases and promotes cell apoptosis [91,92]. Some miRNAs are involved in inhibiting *M. gallisepticum* replication, mainly by upregulating the expression of pro-inflammatory cytokines (such as TNF-α, IL-6, and IL-8), influencing the cell cycle, and activating signaling pathways. Upregulated expression of miR-130b-3p promoted cell proliferation and the inflammatory response through regulating the PI3K/AKT/NF-κB-mediated signaling pathway in *M. gallisepticum*-infected chicken embryo lungs and DF-1 cells by targeting phosphatase and tensin homolog (PTEN) expression in vivo and in vitro, which plays a role in inhibiting cell proliferation and inducing G1 arrest [93]. Furthermore, it was shown that gga-miR-21 negatively regulated *M. gallisepticum* propagation by promoting the proliferation of *M. gallisepticum* infected cells, enhancing the expression of inflammatory cytokines and inhibiting cell apoptosis, which is mediated by activating MAPKs and NF-κB signaling pathways via targeting MAP3K1 in the *M. gallisepticum* infected DF-1 cells [94]. It was found that miR-181a-5p is transported into DF-1 cells through exosomes secreted by CP-II cells and targets PPM1B to activate the TLR2/MYD88/NF-κB signaling pathway, leading to the promotion of cell proliferation, inflammatory response, and inhibition of apoptosis triggered by *M. gallisepticum*-HS infection [95]. Similarly, gga-miR-99a and gga-miR-19a were also found that can trigger the transition from G1 to S and G2 phases of the cell cycle by targeting genes post *M. gallisepticum* infection [96,97]. In addition, some microRNAs promoted cell proliferation and attenuated the inflammatory response and apoptosis to resist *M. gallisepticum* infection. It was found that upon M. gallisepticum infection, Lnc90386 sponged miR-33-5p to negatively regulate the c-Jun N-terminal kinase (JNK) pathway and inhibited *M. gallisepticum* pMGA1.2 expression, promoting cell proliferation and inhibiting inflammatory damage and apoptosis caused by *M. gallisepticum* infection [98].

In chronic respiratory diseases in chickens, aberrant expression of some miRNAs is involved in inhibiting *M. gallisepticum* replication. Host miRNAs affect the replication of pathogenic microorganisms and these microorganisms develop highly complex mechanisms to evade the host immune response. Gga-miR-142-3p, which mainly suppresses the expression of pro-inflammatory cytokines (such as TNF-α, IL-1 and IL-6), influences the cell cycle and activates signaling pathways, thereby influencing *M. gallisepticum* infection [99]. FOXO3 belongs to the O subclass of the forkhead family of transcription factors and plays a crucial role in cell proliferation and apoptosis [100]. It was found that *M. gallisepticum* infection downregulated gga-miR-223 in host cells, which targets FOXO3 to downregulate the expression of cycle genes (CDK1, CDK6, and CCND1) and promotes the expression of apoptosis genes (BIM, FASL, and TRAIL), finally exacerbating *M. gallisepticum* infection [101].

Following *M. gallisepticum* infection, exosomes secreted from *M. gallisepticum*-infected alveolar epithelial cells can cause or exacerbate lung inflammation and significantly increase TNF-α and IL-1β protein levels in DF-1 cells [95]. Gga-miR-193a content increased in exosomes to disturb distal cell proliferation, apoptosis, and cytokine production by targeting the KRAS/ERK signaling pathway [102] and gga-miR-451 was significantly reduced in exosomes, enhancing the inflammatory response in DF-1 cells, although gga-miR-451 expression was highly significantly upregulated in *M. gallisepticum*-infected CP-II cells [90]. In addition, the inflammatory response is critical in modulating the host’s immune response to pathogens such as bacteria, viruses, and parasites but a strong inflammatory immune response is an important cause of CRD. In the early stage of *M. gallisepticum* infection in cells, gga-miR-365-3p was upregulated and activated the JAK/STAT signaling pathway through inhibition of SOCS5 (suppressor of cytokine signaling 5), thus promoting the secretion of inflammatory factors, such as TNF-α and IL-6, and suppressing the expression of pMGA1.2. At the later stage of *M. gallisepticum* infection of cells, SOCS5 downregulates the expression of intracellular gga-miR-365-3p through a negative feedback mechanism, thereby reducing cellular inflammatory damage while promoting mycoplasma adhesion or invasion [71].

Although there is a wealth of research on *M. gallisepticum* pathogenesis at the RNA level, comprehensive summaries are limited. It seems that *M. gallisepticum* utilizes microRNAs to both directly and indirectly influence inflammatory processes, apoptosis, and cell proliferation, altering host cell functions and shaping inflammatory responses to support their survival and proliferation. The host response to *M. gallisepticum* infection is also closely related to miRNAs. Further investigation into the role of miRNAs in the host response to *M. gallisepticum* infection will contribute to a complete understanding of the pathogenesis of *M. gallisepticum* infection at an RNA level.

## 6. The Adaptive Immune Response to *M. gallisepticum*

### 6.1. Mucosal Immunity

*M. gallisepticum* initially colonizes the upper respiratory tract, firmly adheres to the surface of epithelial cells to resist host clearance from mucosal epithelial cell cilia and phagocytosis by phagocytic cells, and then spreads to the lower respiratory trachea, causing bronchitis, airsacculitis, and pneumonia [33]. Therefore, the immunity of the respiratory trachea is crucial to inhibit *M. gallisepticum* infection. The respiratory immune system is associated with various structures connected to the mucosal surface that is exposed to polluted air, including the Harderian gland (HG), conjunctiva-associated lymphoid tissue (CALT), paranasal glands (PG), and bronchus-associated lymphoid tissue (BALT) [103]. They are functionally important components of local immunity, especially in the upper airways.

Although chickens are genetically deficient in lymph nodes, infection of chickens with *M. gallisepticum* strongly activates the tracheal mucosal immune response, causing inflammatory lesions in local tissues; the severity of immune damage is age-related [104]. The different degrees of damage caused by *M. gallisepticum* infection are marked by the development of thickened and debilitated mucosal epithelium due to the large number of lymphocytes, histiocytes, and plasma cells infiltrating the lamina propria, accompanied by a small number of heterophils diffusely distributed in the epithelium and luminal exudate [10]. Thus, the diffuse tertiary lymphoid tissues along the respiratory trachea are involved in local immune response as well as the pathogenesis of *M. gallisepticum* infection.

**Figure 3:**
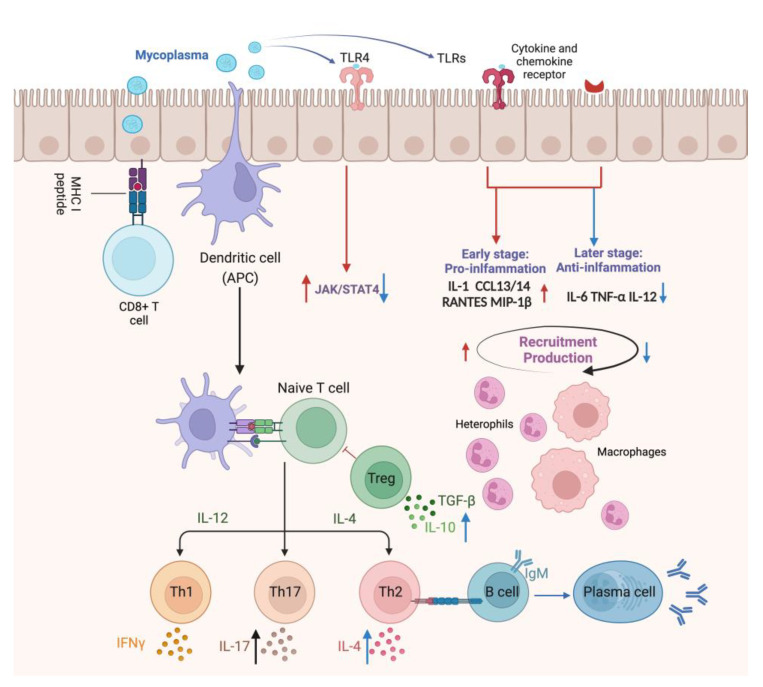
The immune response of the tracheal mucosa to *M. gallisepticum* infection in the respiratory tract. The red arrows indicate the modulation of early-stage immune mechanisms initiated upon infection, highlighting the induction of inflammation and recruitment of immune cells to the site of infection. *M. gallisepticum* attaches and colonizes the airway epithelium and is recognized by epithelial TLRs, initiating a TLR-mediated signaling pathway that regulates the expression of pro-inflammatory factors, including IL-1, CXCL13, CXCL14, RANTES, and MIP-1β, followed by the recruitment of macrophages and inflammatory cells to the local tissue. These recruited immune cells release inflammatory mediators to enhance host response and the secreted chemokines recruit naive B and T lymphocytes to the local tissue, where they are activated for subsequent adaptive immune responses. Conversely, the blue arrows illustrate the late stage of infection when *M. gallisepticum* acts to suppress inflammatory mediators and modulate the immune response, potentially suppressing inflammatory mediators such as IL-6 and TNF-α, influencing Th1 immune responses through the modulation of JAK/STAT4 pathway and cytokines correlated with cell-mediated immunity, such as IL-2 and IL-4, which prevents the host immune system from clearing the pathogen prematurely.

It was reported that there are large numbers of B cells and CD4^+^ and CD8^+^ T cells infiltrating the tracheal mucosa of chickens post *M. gallisepticum* infection, as shown in Figure 3. In five-week-old birds infected with *M. gallisepticum* (R_low_), B cells were found to be the major lymphocyte population infiltrating the mucosa from one day after infection [10]. In another study, B cells were detected three weeks after infection with the AP3AS strain of *M. gallisepticum* in eight-week-old birds and only CD8^+^ and CD4^+^ T cells were observed in the first week [11]. Recently, it was found that B cell recruitment into the mucosa in mature birds occurred later than that in young birds and is generally detectable two weeks after infection [3]. Birds under four weeks of age likely have a stronger immune response and more severe damage than mature birds, with greater concentrations of *M. gallisepticum* in the trachea, which may be related to the maturity of the immune system [104], especially BALT, whose structure, morphology, and ability to perform defense functions in birds are largely age-dependent. Mature BALT is covered with a layer of fragile epithelium called follicle-associated epithelium (FAE), which harbors numerous lymphocytes [105,106]. These tertiary lymphoid tissues play a major role in the tracheal mucosal immune response. B lymphocytes were not detected until 3 weeks after infection and their appearance coincided with a decrease in the concentration of mycoplasma DNA detectable in the trachea [11]. In general, B cells should appear in local inflammatory sites; more efforts will be required to instigate the involvement of immune cells in tissues with *M. gallisepticum* infection. 

### 6.2. Cell-Mediated Immunity

The cell-mediated immune response to *M. gallisepticum* in the tracheal mucosa of vaccinated and unvaccinated chickens during the acute and chronic stages of the disease plays a crucial role in clearing the pathogen. Previous studies have used immunohistochemistry to determine the number of lymphocytes in the upper, middle, and lower trachea of chickens [29]. It was found that both CD8^+^ and CD4^+^ cells were observed in the first week of *M. gallisepticum* infection, notably with an elevated prevalence of CD8^+^ cells and the formation of follicular aggregates within the tracheal mucosa. However, the vaccinated birds did not have significantly greater numbers of CD4^+^ or CD8^+^ lymphocytes in their tracheal mucosa [10,107]. In unvaccinated chicken, CD8^+^ cells peaked at 1 week and then declined significantly in the tracheal mucosa [3] and similar changes in CD8^+^ cell number and distribution were observed in the tracheal mucosa of infected turkeys [107]. However, these cells were shown to be CD8^+^TCR^−^ cells by double immunohistochemical staining of TCR and CD8 and are likely to be NK or NK-like cells, which are involved in the initial, possibly non-specific, inflammatory response and may be related to the pathogenesis of *M. gallisepticum* in the trachea [11], as increased expression of various chemokines and proinflammatory cytokines was observed one week post-M. *gallisepticum* infection [4]. The numbers of CD8^+^TCR^+^ cells increased over 6 weeks after infection [11], suggesting a subsequent cytotoxic T-cell response to *M. gallisepticum* infection.

The number of CD4^+^ cells was low in the first two weeks and then remained high from the third week post-infection. The proportion of CD4^+^TCRαβ1^+^ cells increased over the first 3 weeks and then subsequently decreased, while the proportion of CD4^+^TCRαβ2^+^ cells constantly increased during the 6-week experimental period [11]. The shift in CD4^+^ cells in the trachea from CD4^+^TCRαβ2^+^ to CD4^+^TCRαβ1^+^ during the infection suggests a change in T-cell immunity in the trachea after infection.

## 7. Escape from the Adaptive Immune Response in Trachea

Local immunity for tracheal mucosa plays an important role in preventing mycoplasma attachment to respiratory epithelia. Unvaccinated chickens had several defined B-cell clusters with many interspersed CD4^+^ cells [10] and these B cells are Th-dependent and mainly secrete lgA. The number of IgA^+^ cells is high in unvaccinated chickens after infection but does not contribute to accelerated pathogen clearance and recovery from infection, probably due to the immune escape of the pathogen and the fact that mucosal immunity exists mainly on the mucosal surface and covers a small area [10]. In contrast, GT5-vaccinated chickens were found to have lower numbers of infiltrating lymphocytes, with more B-cells organized in a typical follicular pattern one week after infection. Chickens vaccinated with ts304 showed a similar situation, with no CD3^+^, CD4^+^, or CD8^+^ cells detected in the tracheal mucosa two weeks after challenge at 60 weeks of age [3]. B cells without help from CD4^+^ cells are commonly Th-independent and mainly secrete IgM, which promotes *M. gallisepticum* clearance and reduces the severity of the damage. Several vaccines, such as ts-11, GT5, and ts304, have been shown to effectively prevent the development of the disease in birds after challenge [104]. 

In general, the regulation of mucosal immunity by *M. gallisepticum* includes the following. First, *M. gallisepticum* infection leads to damage of mucosal epithelial cells, which inhibits the generation and impaired function of mucosal immunity. Second, the different types of antibodies produced by B cells and the degree of lymphocyte infiltration may have an impact on mucosal immunity. Third, pMGA phenotypic variation in *M. gallisepticum* occurs by selection pressures induced by antibodies and this might be an essential tactic employed by the pathogen for immune evasion [108]. Furthermore, CysP (cysteine protease) is involved in the cleavage of IgG by *M. synoviae* and *M. gallisepticum*. The IgG cleavage pattern is similar to that of papain, a cysteine protease that cleaves IgG into Fab and Fc, but the effect of this on immune escape is not yet known [109]. CD8^+^ cell depletion may be a result of immune dysregulation caused by *M. gallisepticum*, which contributes to their replication and survival [8]. Similar signs of immune dysregulation have been found in recent studies in the bursa of Fabricius [8,110]. Immune dysregulation after *M. gallisepticum* infection may be related to Th cell differentiation and proliferation, which could be cross-regulated by the effector cytokines produced by other Th subsets. CD4^+^ Treg cells secreting IFN-γ and IL-17 can dampen Th2 responses, including the IL-13 response and the serum antibody response to chronic infections with *M. pulmonis* [111]. Moreover, an upregulation of IFN-γ and IL-17 was also found two weeks after mycoplasma infection and at the same time, the downregulation of IL-2 also corroborates the suppression of the cellular immune response [3]. However, the exact mechanism underlying mycoplasma-induced immune compromise requires further investigation.

Recent studies suggest that *M. gallisepticum* induced immune dysregulation may be associated with TLR4-mediated regulation of the JAK/STAT signaling pathway, exerting opposite effects of promoting or inhibiting Th subpopulation polarization at different times of infection [75]. It was shown that on day 3 after *M. gallisepticum* infection, TLR4 mediated the activation of IL-12Rβ2 and TYK2, leading to STAT4 phosphorylation and the release of proinflammatory factors. However, the expression of these genes tended to decrease and the activation of the JAK/STAT signaling pathway was inhibited on days 5 and 7 post-infection, suggesting that *M. gallisepticum* regulates the transition from the Th1 to Th2 type of adaptive immunity. This may explain the depletion of CD8+ cells in the early phase of *M. gallisepticum* infection in the trachea.

## 8. Immune Organ Damage Caused by *M. gallisepticum* Infection 

Bursa of Fabricius (BF) is the central immune organ of birds and the primary site for the generation, development, and maturation of B lymphocytes, which play a crucial role in host immunity against *M. gallisepticum* infection [112]. Previous studies have demonstrated that *M. gallisepticum* infection impairs the structural integrity and T cell-mediated immunity to clear pathogens in BF within 48 h post-*M. gallisepticum* infection [113]. The number of CD8^+^ lymphocytes significantly decreased in chicken BF at day 7 post-*M. gallisepticum* infection and apoptosis and oxygen species (ROS) increased in BF tissues after *M. gallisepticum* infection [8]. Additionally, the mRNA and protein expression levels of autophagy-related genes were significantly reduced and the level of the mTOR gene, as evaluated, showed that autophagy was inhibited [110]. Autophagy has been known as the cell homeostatic mechanism removing pathogens or damaged organelles and protecting cells by reducing DAMPs or PAMPs [114]. Studies have shown that baicalin upregulates autophagy levels, thereby attenuating tissue damage caused by *M. gallisepticum* and reducing its load [110].

The thymus is a primary lymphoid organ and a critical site for the generation, differentiation, and maturation of T cells. A thymic injury may lead to severe immunocompromised pathology [115]. Recently, studies have gradually revealed the underlying molecular mechanisms by which *M. gallisepticum* infection induces immune damage to the thymus. It was found that there was structural damage in the thymus of chickens with *M. gallisepticum* infection, including lymphocytosis, discrete cell arrangement, and accumulated nuclear debris [116]. Furthermore, the number of CD8^+^ lymphocytes has significantly decreased in the *M. gallisepticum* infection group and typical apoptotic features including mitochondrial swelling, chromatin shrinkage and condensation, and DNA fragmentation were found as examined by ultrastructure observation and analysis [117]. Meanwhile, *M. gallisepticum* infection induced oxidative stress and mitochondrial dynamic imbalance, triggered the inflammatory response through the TLR-2/MyD88/NF-κB signaling pathway, activated the NLRP3 inflammasome, and induced the secretion of IL-1β [117,118], which is closely related to cell death, immune disorders, and inflammatory diseases [50,119,120]. For example, inflammasome activation in COVID-19-infected macrophages drives a cytokine storm and inflammatory damage and inhibitors of NLRP3 could attenuate COVID-19 pathology [121].

It was reported that *M. gallisepticum* infection could induce autophagy in RAW264.7 cells through the extracellular regulated protein kinase (ERK) signaling pathway [61], which can decrease the accumulation of DAMPs or PAMPs and reduce mitochondrial damage [122]. On the contrary, autophagy was inhibited in primary immune organs after *M. gallisepticum* infection [110,118]. Andrographolide (AG) attenuates the inflammatory response by inhibiting the PI3K/Akt pathway to regulate autophagy in HD11 cells [123]. Notably, the antioxidant effect of Baicalin can efficiently alleviate oxidative stress and apoptosis in the thymus after *M. gallisepticum* infection through the Nrf2/HO-1 signaling axis [116]. Further studies will be needed to explore the molecular mechanism of autophagy associated with *M. gallisepticum* infection, which might be of help to the development of effective strategies for the prevention and control of *M. gallisepticum* infection.

## 9. Prevention of *M. gallisepticum*

With the increase in antimicrobial resistance and the diminishing efficacy of antibiotics for *M. gallisepticum* control, the improvement in existing vaccines and the development of new ones have become increasingly important. Both inactivated and live vaccines have been widely used for *M. gallisepticum* prevention. Inactivated vaccines typically induce a high level of humoral immune response and lower the risk of reversion to a pathogenic form of the vaccine. Live vaccines, such as the F strain [124] and ts-11 strain [125], have been proven effective in preventing air sac infection and respiratory diseases caused by *M. gallisepticum* and in reducing losses in production performance. In general, live vaccines derived from F strains induce higher levels of antibodies against *M. gallisepticum* than those derived from ts-11 strains [126]. With technological advancements, new generations of *M. gallisepticum* vaccine candidates like GT5 [127], MG 7 [128], K-strain [129], and ts-304 [130] have been researched to address concerns about existing vaccine strains. GT5 and MG 7 originated from the virulent R_low_ strain, while the K-strain has shown effectiveness in the upper respiratory tract for about five months [131], protecting chickens from tracheal lesions. The ts-304, a variant of the ts-11 strain, offers a higher protection [132].

Genetically engineered vaccines, such as recombinant vaccines, are increasingly recognized as crucial in combating *M. gallisepticum* infections. For example, a recombinant Fowl Pox vector has been developed to express *M. gallisepticum* 40k and mgc genes, offering a new avenue for vaccination strategies [133]. Additionally, the use of recombinant adenovirus vectors for expressing the S1 spike glycoprotein of the infectious bronchitis virus (IBV) and the TM-1 protein of *M. gallisepticum* in HEK293 cells has shown promise in reducing clinical signs and lesions associated with these infections [134]. Recent studies have focused on designing multi-epitope vaccines using immunoinformatics, targeting antigenic proteins of *M. gallisepticum* [135,136]. These candidate vaccines, comprising cytotoxic T-cell (CTL), helper T-cell (HTL), and B-cell epitopes, show promising results in structural stability, specificity, and immunogenic response, predicting effective binding with chicken Toll-like receptors and indicating strong immune induction potential but which need to be validated in animal experiments. In addition to traditional bacterial, yeast, and mammalian expression systems, future vaccine development includes the use of plants as biofactories for vaccine production [137]. A notable example is the stable expression of the TM-1 gene of *M. gallisepticum* in wheat seed tissues, providing a safe, scalable, and cost-effective vaccine option. This plant-made vaccine, when orally delivered, elicited an effective antibody response in chickens, comparable to commercially available inactivated vaccines against CRD [138]. These vaccines, as well as those developed in the future, are expected to elicit a strong immune response and provide a safe, highly specific, and stable preventive option.

## 10. Conclusions

Avian mycoplasmas tactically evade innate and adaptive immune responses via different mechanisms. It is clear that *M. gallisepticum* induces an inflammatory response in the host, causing immune damage. Currently, it is known that *M. gallisepticum* infection of the airway elicits a strong inflammatory response, recruiting lymphocytes, macrophages, and heterophils to the mucosal lamina propria, which produce a large number of cytokines and chemokines that modulate the immune response while causing pathological damage. In addition, *M. gallisepticum* infection can regulate cellular microRNAs to facilitate its replication and survival. As *M. gallisepticum* can reach different organs of the whole body after infection, causing inflammatory responses and immune damage, it seems that the pathogen can successfully evade the host’s immune response. However, the mechanism by which hosts combat *M. gallisepticum* or the latter evades the former seems much more complicated than previously thought. Further studies will be required to investigate the mechanism that regulates *M. gallisepticum* infection, disease progression, or host responses to *M. gallisepticum* infection. No doubt, the elucidation of the pathogenesis of *M. gallisepticum* infection will be of great help to the development of effective strategies for the prevention and control of *M. gallisepticum* infection.
